# Oral octreotide capsules for the treatment of acromegaly: comparison of 2 phase 3 trial results

**DOI:** 10.1007/s11102-021-01163-2

**Published:** 2021-06-25

**Authors:** Artak Labadzhyan, L B Nachtigall, M Fleseriu, M B Gordon, M Molitch, L Kennedy, S L Samson, Y Greenman, N Biermasz, M Bolanowski, A Haviv, W Ludlam, G Patou, C J Strasburger

**Affiliations:** 1 Cedars-Sinai Medical Center, Los Angeles, CA 90048 USA; 2 MGH Neuroendocrine and Pituitary Center, Chestnut Hill, MA USA; 3 Pituitary Center, Oregon Health & Sciences University, Portland, OR USA; 4 Allegheny Neuroendocrinology Center, Allegheny General Hospital, Pittsburgh, PA USA; 5 Northwestern University, Chicago, IL USA; 6 Cleveland Clinic Foundation, Cleveland, OH USA; 7 Mayo Clinic, Jacksonville, FL USA; 8 Sourasky Medical Center and Tel Aviv University, Tel Aviv, Israel; 9 Leiden University Medical Center, Leiden, The Netherlands; 10 Wroclaw Medical University, Wroclaw, Poland; 11 Chiasma, Inc., Needham, MA USA; 12 Charite Universitatsmedizin, Berlin, Germany

**Keywords:** Oral octreotide, Acromegaly, IGF-I, Somatostatin receptor ligands, Somatostatin analogs, Growth hormone

## Abstract

**Purpose:**

Results are presented from 2 to 3 trials investigating oral octreotide capsules (OOC) as an alternative to injectable somatostatin receptor ligands (iSRLs) in the treatment of acromegaly.

**Methods:**

CH-ACM-01 was an open-label trial (N = 155) and CHIASMA OPTIMAL was a double-blind placebo-controlled (DPC) trial (N = 56), both investigating OOC as maintenance therapy for patients with acromegaly who were biochemical responders receiving iSRLs.

**Results:**

Baseline characteristics in both trials reflected those expected of patients with acromegaly responding to treatment and were similar between trials, despite differences in inclusion criteria. OOC demonstrated a consistent degree of biochemical response across trials, with 65% of patients in CH-ACM-01 maintaining response during the core period and 64% of patients in CHIASMA OPTIMAL at the end of the DPC. Mean insulin-like growth factor I (IGF-I) levels remained within inclusion criteria at the end of treatment in both trials. Of 110 patients entering the fixed-dose phase in CH-ACM-01, 80% maintained or improved acromegaly symptoms from baseline to the end of treatment. Over 85% of patients in both trials elected to continue into the extension phases. OOC were found to be well tolerated across both trials, and no dose-related adverse events were observed.

**Conclusions:**

OOC demonstrated remarkably consistent results for biochemical response, durability of response, and preference to continue with oral treatment across these 2 complementary landmark phase 3 trials, despite differences in the design of each.

**Trial registration** NCT03252353 (August 2017), NCT01412424 (August 2011).

## Introduction

Acromegaly is characterized by excessive circulating levels of growth hormone (GH) and insulin-like growth factor I (IGF-I), usually resulting from a GH-secreting pituitary adenoma [1–5]. Treatment for acromegaly is aimed at normalizing GH and IGF-I levels, shrinking tumors, amelioration of symptoms, improving quality of life, and reducing mortality with as few side effects as possible [1, 6, 7]. Pituitary surgery is the primary therapy for most patients with acromegaly [1, 2]. For patients unwilling or unable to undergo surgery, or in patients with persistent or recurrent disease, medical therapy is indicated [2, 8, 9]. Medical therapy includes somatostatin receptor ligands (SRLs), GH receptor antagonists, and dopamine agonists in single or combination therapy [10, 11]. Long-acting injectable SRLs (iSRLs) are a cornerstone of medical treatment in acromegaly and have demonstrated efficacy in attenuating serum GH and IGF-I levels, reducing tumor size, and improving symptoms [12]. However, only 56% of patients expressed satisfaction with current injectable treatments in 1 study [13]. Patients receiving iSRLs through deep tissue injection often report injection site pain, nodules, bruising, inflammation, and scarring as well as anxiety, frustration, and loss of independence. Additionally, many patients report missing work in order to receive injections and worsening of symptoms toward the end of the dosing interval [5, 9]. The Acromegaly Treatment Satisfaction Questionnaire (Acro-TSQ) is a scale that was developed to provide a complete assessment of the disease and treatment burdens associated with acromegaly and for tracking the overall efficacy and unmet needs of new treatments for patients with acromegaly [9, 14]. In 1 analysis employing the Acro-TSQ, two-thirds of patients who were biochemically responding to iSRLs reported ongoing acromegaly symptoms, with > 80% experiencing those symptoms all the time [15]. Avoiding injections, potentially by substituting an oral formulation, was mentioned as a top preference for new acromegaly treatments among 85% of respondents [13].

To address the patient burden associated with iSRLs, an oral formulation of octreotide has been developed. Oral octreotide capsules (OOC) combine octreotide with proprietary excipients (Transient Permeability Enhancer®) to form an oily suspension of hydrophilic particles in a lipophilic medium [16, 17]. OOC are the first approved oral SRL for acromegaly in the United States and are indicated for long-term maintenance treatment in patients with acromegaly who have responded to and tolerated treatment with octreotide or lanreotide. Results of 2 complementary but distinct phase 3 multicenter trials examining the safety and efficacy of OOC have been reported. The first (CH-ACM-01) was an open-label, dose titration trial that included a large number of patients with acromegaly who were previously receiving iSRLs; the results from that trial have been reported previously [18]. The second trial, OOC-ACM-303 (CHIASMA OPTIMAL), was a randomized, double-blind, placebo-controlled (DPC) trial that served as the basis for US Food and Drug Administration approval of OOC as a long-term maintenance treatment in patients with acromegaly in whom prior treatment with SRLs has been shown to be effective and tolerated [19]. The objective of this report is to provide a more complete understanding of the safety and efficacy of OOC in patients with acromegaly, by comparing and contrasting the results obtained from the above trials with different designs.

## Comparison of methods

**Fig. 1:**
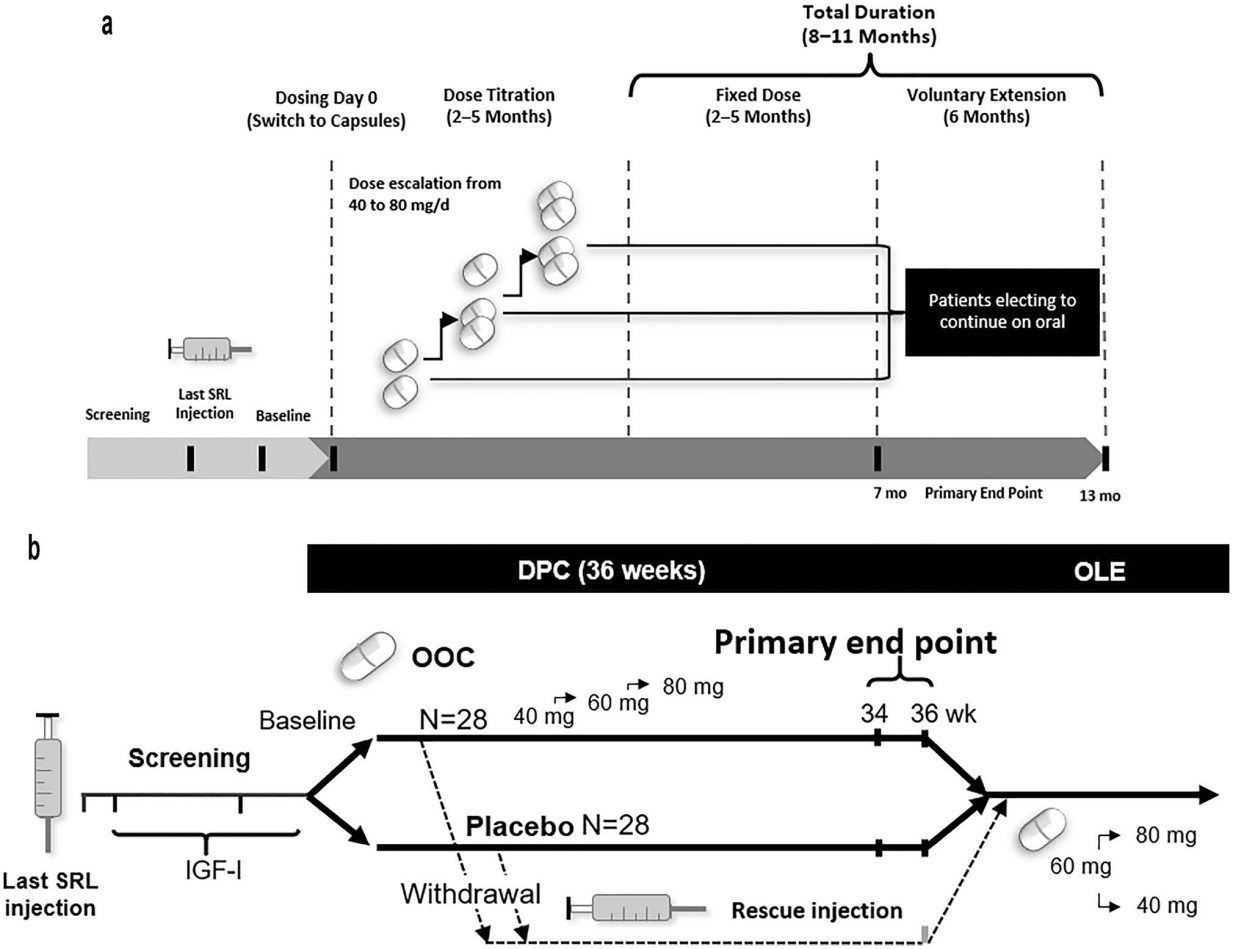
Study design for phase 3 trials. The trial design for CH-ACM-01 (**a**) was open label and included an optional extension period while CHIASMA OPTIMAL (**b**) was DPC with an OLE. *DPC* double-blind, placebo controlled, *OLE* open-label extension, *OOC* oral octreotide capsules, *SRL* somatostatin receptor ligand

Key differences in the 2 previously described protocols [18, 19] are summarized in Table 1. The CHIASMA OPTIMAL trial required a more stringent inclusion cutoff for IGF-I (≤ 1.0 × upper limit of normal [ULN]), and the CHIASMA OPTIMAL trial included a placebo arm. The CH-ACM-01 trial had an inclusion cutoff of IGF-I < 1.3 × ULN and was open label. Primary endpoints varied, with both reflecting the different levels of biochemical response for entry criteria, as measured by IGF-I levels, with CHIASMA OPTIMAL using an average of 2 visits compared to a single measurement for the CH-ACM-01. The timing of baseline IGF-I measurements related to last dose of iSRL varied between the 2 trials, with the baseline measurement taken within 4 weeks of the last SRL injection for CH-ACM-01, and the baseline measurements being taken between 4 and 8 weeks from the last injection based on the prior iSRL dosing interval for a given patient for CHIASMA OPTIMAL (Fig. 1; Table 1). The imputation methods also differed between the 2 trials; CH-ACM-01 used last observation carried forward (LOCF), and CHIASMA OPTIMAL trial used worst observation carried forward (WOCF), or nonresponse imputation in the analysis of the primary endpoint (i.e., any patient who did not complete the full 9 months of treatment was considered a treatment failure regardless of the reason). For assessment of acromegaly symptom-related data, the Acromegaly Index of Severity (AIS) was used for CH-ACM-01, while for CHIASMA OPTIMAL, new or worsening signs and/or symptoms of acromegaly were monitored via adverse event (AE) reporting of AEs of special interest (AESIs; such as headache, perspiration, joint pain, fatigue, and soft tissue swelling, increases in blood pressure, and increases or decreases in blood sugar).

Both trials had an optional extension phase. There were no dose restrictions for continuation into the 6-month extension period in CH-ACM-01. In CHIASMA OPTIMAL, patients could enter the open-label extension (OLE) if they completed 36 weeks on oral treatment (oral or placebo) or if they met the predefined withdrawal criteria (i.e., IGF-I ≥ 1.3 × ULN and exacerbation of acromegaly clinical signs or symptoms). Upon entering the OLE phase, the starting dose for all patients was 60 mg and was titrated based on IGF-I levels and/or signs and symptoms of acromegaly.

**Table 1:** Comparison of protocols of CH-ACM-01 and CHIASMA OPTIMAL trials

	CH-ACM-01	CHIASMA OPTIMAL
Study design	Phase 3, multicenter, open-label, baseline-controlled trial	Phase 3, multicenter, randomized, DPC trial
Patient population	• Patients with response to iSRL (defined as IGF-I < 1.3 × ULN for age and integrated GH response over 2 h of < 2.5 ng/mL) • N = 155	• Patients with response to iSRL treatment (defined as IGF-I ≤ 1.0 × ULN based on average of 2 screening assessments, 1 within 2 weeks of baseline) • N = 56 • Randomized 1:1 • 28 OOC, 28 placebo
Patient stratification	N/A	Patients were stratified based on prior iSRL dose (low vs. mid/high)^a^
Duration of treatment	• Core treatment phase: up to 7 mo (dose escalation phase of 2–5 mo and fixed-dose phase of 2–5 mo) • Voluntary extension (open-label, fixed dose): 6 mo	• DPC phase: 36 wk • Voluntary extension (open label, 60 mg starting dose with titration up or down allowed): 1 y (with multiple extensions of 1 y each)
Criteria for reversion to iSRLs	• Per investigator discretion, patients could revert to iSRL therapy any time during the trial for efficacy or safety reasons	• Predefined withdrawal criteria were IGF-I ≥ 1.3 × ULN for 2 consecutive visits and exacerbation of acromegaly clinical signs/symptoms while treated with 80 mg/d dose for ≥ 2 wk • Per investigator discretion, patients could revert to iSRL therapy any time during the trial for efficacy or safety reasons
Dose escalation recommendations	• Dose escalation: started at 40 mg/d and titrated to 60 mg/d and 80 mg/d • Based on measurement of each participant’s circulating IGF-I levels (> 20% increase over prior levels in 2 successive visits) AND/OR • Emergence of acromegaly-related symptoms	• Dose escalation: started at 40 mg/d and titrated to 60 mg/d and 80 mg/d • Per investigator’s discretion based on any 1 of the following recommendations: o Significantly increased IGF-I levels compared to baseline defined as IGF-I increase by ≥ 30% to > 1.0 × ULN o IGF-I levels > 1.0 × ULN for 2 consecutive visits o New or worsening signs/symptoms of acromegaly
Timing of baseline measurements/treatment initiation	• Baseline measurements were taken anytime within 4 wk from last SRL injection • OOC was administered no less than 1 mo following the last SRL injection	• Baseline measurements were taken 4 to 8 wk from last SRL injection depending on the patient’s previous dosing interval • OOC was started on the day of the next anticipated injection (± 3 d)
Primary endpoint	Maintenance of response based on IGF-I and GH levels	Maintenance of response based on IGF-I levels
Timepoint	• Single timepoint at the end of the core treatment period (≥ 7 mo)	• Average of 2 timepoints (week 34 and 36)
Threshold	• IGF-I < 1.3 × ULN and GH < 2.5 ng/mL	• IGF-I ≤ 1.0 × ULN
Imputation method for missing data used in analysis of primary endpoint	LOCF	Nonresponse imputation (WOCF)
Secondary and exploratory endpoints	Response levels at the end of treatment based on IGF-I and/or GH levels	• Integrated GH response, measured every 30 min for 5 timepoints; (< 2.5 ng/mL) at week 36 • Time to loss of IGF-I response (> 1 × ULN or ≥ 1.3 × ULN) • Proportion reverting to iSRL
Patient-reported outcomes	Treatment satisfaction via TSQM (validated)	None
Symptom assessments	*Maintenance, improvement, or worsening* via regular AIS assessment (unvalidated)	*New or worsening* AESIs (signs/symptoms of acromegaly such as headache, perspiration, joint pain, fatigue, soft tissue swelling, hypertension, dysglycemia)
Key differences in inclusion/exclusion criteria	• Allowed patients who received conventional radiotherapy > 10 y and stereotactic > 5 y prior to screening • Only allowed patients on monthly or more frequent dosing of iSRLs	• No conventional or stereotactic radiotherapy any time in the past allowed • Allowed patients receiving iSRLs at any approved dose including extended interval (e.g., lanreotide 120 mg every 6 or 8 wk)
Key criteria for entry into extension	• Patients must have completed the full duration of the core treatment and the end-of-treatment visit according to the protocol • Patients had levels of IGF-I that were normalized, falling, or had returned to baseline levels during at least the prior 2 successive clinic visits	• Patients must have completed the full 36-wk duration of the DPC period on assigned trial medication OR • Patients met the predefined withdrawal criteria during the DPC period and completed follow-up per protocol through week 36 of DPC period

*AE* adverse event, *AESI* adverse event of special interest, *AIS* Acromegaly Index of Severity, *DPC* double-blind, placebo-controlled, *GH* growth hormone, *IGF-I* insulin-like growth factor I, *iSRL* injectable somatostatin receptor ligand, *LOCF* last observation carried forward, *OOC* oral octreotide capsules, *SRL* somatostatin receptor ligand, *TSQM* Treatment Satisfaction Questionnaire Medication, *ULN* upper limit of normal, *WOCF* worst observation carried forward

^a^High SRL dose: lanreotide 120 mg every 4 wk, octreotide 30 mg every 4 wk; mid SRL dose: lanreotide 90 mg every 4 wk or 120 mg every 6 wk, octreotide 20 mg every 4 wk; low SRL dose: lanreotide 60 mg every 4 wk or 120 mg every 8 wk, octreotide 10 mg every 4 wk

## Results

A total of 235 individuals were screened for CH-ACM-01, of whom 80 (34%) failed screening and 155 were enrolled. For CHIASMA OPTIMAL, 119 individuals were screened; 63 (53%) failed screening and 56 of were enrolled into the trial, 28 of whom were randomized to OOC. Baseline characteristics are described in Table 2. The baseline disease characteristics of patients enrolled in both trials were very similar despite the difference in biochemical values due to varying inclusion criteria (IGF-I < 1.3 × ULN for the CH-ACM-01 trial and IGF-I ≤ 1.0 × ULN for CHIASMA OPTIMAL).

**Table 2:** Baseline characteristics of CH-ACM-01 and CHIASMA OPTIMAL trials

	CH-ACM-01 (N = 155)	CHIASMA OPTIMAL: OOC Group (n = 28)^a^
Symptom burden at baseline		
≥ 1 symptom, n (%)	125 (81)	23 (82.1)
≥ 2 symptoms, n (%)	95 (61)	18 (64.3)
≥ 3 symptoms, n (%)	67 (43)	10 (35.7)
Mean baseline IGF-I levels, × ULN (SD)	0.94 (0.25)	0.8 (0.16)
Biochemical control at screening (cutoff)	100% (IGF-I < 1.3 × ULN)	100% (IGF-I ≤ 1.0 × ULN)
Biochemical control at baseline, n (%)		
IGF-I ≤ 1.0 × ULN	95 (61)	27 (96.4)
IGF-I > 1 to < 1.3 × ULN	42 (27)	1 (3.6)
IGF-I ≥ 1.3 × ULN	18 (12)	0
Duration of acromegaly, n (%)		
< 10 y	74 (47.7)	15 (53.6)
10–20 y	53 (34.2)	8 (28.6)
≥ 20 y	28 (18.1)	5 (17.9)
Prior SRL treatment, n (%)		
Octreotide low dose^b,c^	64 (41.3)	3 (10.7)
Octreotide mid/high dose^b,c^	33 (21.3)	16 (57.1)
Lanreotide low/mid dose^b,c^	25 (16.1)	4 (14.3)
Lanreotide high dose^b,c^	33 (21.3)	5 (17.9)
Baseline weight kg (SD)	86.25 (19.305)	83.4 (17.22)
BMI kg/m^2^ (SD)	NC	29.1 (6.26)
Diabetes mellitus, n (%)	29 (18.7)	6 (21.4)

*BMI* body mass index, *GH* growth hormone, *IGF-I* insulin-like growth factor I, *NC* not calculated, *OOC* oral octreotide capsules, *SRL* somatostatin receptor ligand, *ULN* upper limit of normal

^a^Data shown from the OOC group only in CHIASMA OPTIMAL. Placebo data were reported previously [19]

^b^Doses are defined as follows: For CH-ACM-01: octreotide low dose was considered to be 10 or 20 mg every 4 wk; octreotide mid/high was considered to be 30, 40, or 60 mg every 3–4 wk; lanreotide low/mid was 30, 60, or 90 mg every 3–4 wk; and lanreotide high was 120 mg every 3–4 wk

^c^For CHIASMA OPTIMAL, octreotide low dose was considered to be 10 mg every 4 wk; octreotide mid/high were considered to be 20 or 30 mg every 4 wk; lanreotide low/mid were considered 60 or 90 mg every 4 weeks or 120 mg every 6–8 weeks; lanreotide high was considered to be 120 mg every 4 weeks

For both trials, > 80% of patients reported active acromegaly symptoms at baseline despite long-term treatment with iSRLs (injectable long-acting forms of either octreotide or lanreotide) [18, 19]. In CH-ACM-01, at baseline, 81% of patients reported ≥ 1 acromegaly symptom, 61% had ≥ 2, and 43% had ≥ 3 (Table 2). In the OOC group of CHIASMA OPTIMAL, at baseline, 82% of patients had ≥ 1 symptom, 64% had ≥ 2, and 36% had ≥ 3. For the placebo group, 86% had ≥ 1 symptom, 68% had ≥ 2, and 50% had ≥ 3 (Table 2).

Eligibility in the CH-ACM-01 trial was based on a screening visit prior to the baseline visit, and for CHIASMA OPTIMAL, baseline IGF-I values were derived as an average of the second screening value and the baseline visit. Of interest, some patients had lost biochemical response between screening and the baseline visit in both trials: 12% of patients in CH-ACM-01 and 11% of patients in CHIASMA OPTIMAL no longer had IGF-I levels below the screening cutoff, suggesting variability in IGF-I levels in patients with biochemically controlled acromegaly (Table 2).

### Efficacy of OOC

Both trials demonstrated the effectiveness of OOC in all prespecified analyses. Using LOCF imputation, 65% of patients in the CH-ACM-01 trial maintained response during the core phase (response defined as IGF-I ≤ 1.3 × ULN), and 64% of patients in the OOC group of the CHIASMA OPTIMAL trial maintained response at the end of the DPC period (response defined as IGF-I ≤ 1.0 × ULN). Using the WOCF imputation, maintenance of response was 53 and 58% in the CH-ACM-01 and CHIASMA OPTIMAL trials, respectively (Table 3) [18, 19]. More than 85% (86 and 90% in the CH-ACM-01 and CHIASMA OPTIMAL trials, respectively) of the patients enrolled in the extension phases of each trial. Efficacy outcomes of the OOC treatment arms of CH-ACM-01 and CHIASMA OPTIMAL trials are summarized in Table 3. Efficacy outcome data for the CHIASMA OPTIMAL placebo group were reported previously [19].

**Table 3:** Efficacy outcomes of CH-ACM-01 and CHIASMA OPTIMAL trials

	CH-ACM-01	CHIASMA OPTIMAL
Primary endpoint (WOCF), %	53 (IGF-I < 1.3 × ULN and GH < 2.5 ng/mL)	58 in OOC group (IGF-I ≤ 1.0 × ULN)
Primary endpoint (LOCF), %	65 (all patients; IGF-I < 1.3 × ULN and GH < 2.5 ng/mL)	64 in OOC group (IGF-I ≤ 1.0 × ULN)
Biochemical response in patients on study drug who entered the fixed-dose period and completed the trial, n/N (%)	82/110 (75)	16/21 (76)
GH response (WOCF, among baseline responders), %	67	78 in OOC group
GH response (LOCF, among baseline responders), %	96 (complete responders only)	96 in OOC group
Sustained response (WOCF, end of dose titration to end of trial), %	85	92 sustained response (no imputation needed)
Completed the trial on study drug, %	66	75
Patients electing to continue into the OLE, %	86 (only completers were eligible to participate in the optional extension)	OOC group • 91 (of patients [n = 19] who completed the trial on treatment)
Mean IGF-I levels at the end of treatment (fixed-dose phase for CH-ACM-01 or DPC for CHIASMA OPTIMAL)	1.04 × ULN	0.97 × ULN for the OOC group
Mean GH levels at the end of treatment (fixed-dose phase for CH-ACM-01 or DPC for CHIASMA OPTIMAL)	0.60 ng/mL	0.6 ng/mL in the OOC group

*DPC* double-blind, placebo-controlled, *GH* growth hormone, *IGF-I* insulin-like growth factor I, *LOCF* last observation carried forward, *OLE* open-label extension, *OOC* oral octreotide capsules, *ULN* upper limit of normal, *WOCF* worst observation carried forward

Post hoc analysis using longitudinal IGF-I and GH measurements were used to provide a time-weighted average (TWA) that included all measurements for CH-ACM-01. TWA is an integrated measure of response over the entirety of the treatment period, which is more clinically relevant because fluctuations of IGF-I (± 30%) are common [20–22]. Single timepoint responder analyses have limited utility in quantifying durability of response [23, 24]. Of the patients who entered the fixed-dose period, 80% completed the core period and were responders using TWA, in comparison to 75% using endpoint analysis. Additionally, 95% of patients maintained response throughout the extension period using TWA analysis versus 85% using endpoint analysis.

### Outcomes by prior injectable dose

In CH-ACM-01, patients previously treated with low-mid doses versus high doses (dosing; Table 2) of long-acting SRLs had a 71.6 and 55.6% response rate at the end of treatment, respectively, but a formal between-group comparison was not performed. In CHIASMA OPTIMAL, maintenance of response was observed in 66.7% (4/6) of patients previously receiving low doses of iSRLs and 54.5% (12/22) of patients on medium-high injected doses. The treatment effect in CHIASMA OPTIMAL was consistent irrespective of prior dose of iSRL (odds ratio, 5.4 in low dose; 5.9 in medium-high dose).

**Fig. 2:**
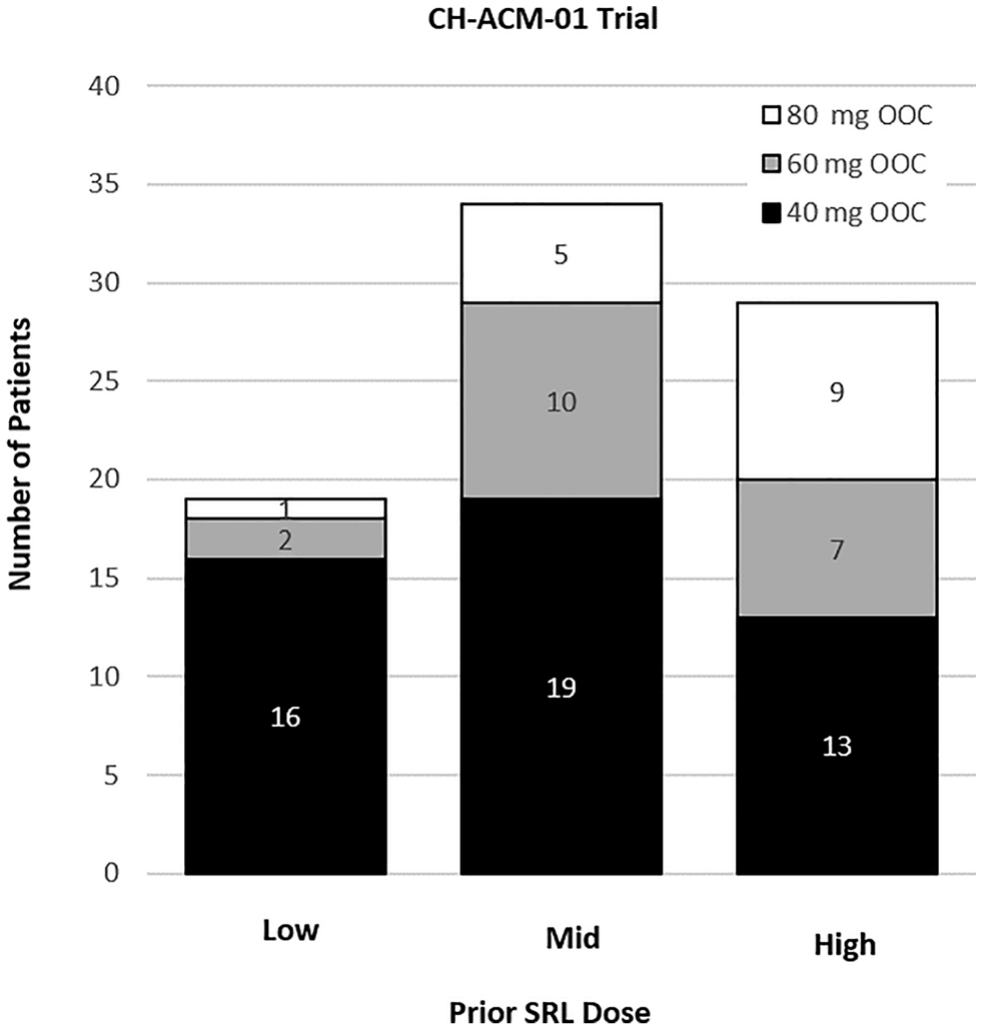
Relationship of Prior iSRL Dose to Final OOC Dose in Responders in CH-ACM-01. For the CH-ACM-01 study, patients previously receiving a low, mid, or high stable dose of iSRL were titrated to a final OOC dose of 40, 60, or 80 mg. *iSRL* injectable somatostatin receptor ligand, *OOC* oral octreotide capsules

Responders during the core treatment period of CH-ACM-01 (n = 82) had final OOC doses of 40 mg (n = 48), 60 mg (n = 19), and 80 mg (n = 15). Breakdown by prior iSRL dose did not indicate a clear relationship with OOC dose in CH-ACM-01 (Fig. 2). This was not assessed in CHIASMA OPTIMAL because it is difficult to extrapolate dose correlations from CHIASMA OPTIMAL in the same manner as CH-ACM-01 owing to the limited sample size.

### Acromegaly symptoms

80% of patients entering the fixed-dose phase improved or maintained acromegaly symptoms (36% maintained; 44% improved) at the end of the CH-ACM-01 trial. At the end of treatment, 80% of patients reported ≥ 1 acromegaly symptom, 56% had ≥ 2, and 38% had ≥ 3. Acromegaly symptoms were not an efficacy endpoint for the CHIASMA OPTIMAL trial; however, AESIs were observed more frequently in patients receiving placebo than those receiving OOC (92.9% vs. 53.6%), indicating the emergence of acromegaly signs and symptoms while off treatment. The most common AESIs observed were arthralgia, hyperhidrosis, fatigue, carpal tunnel syndrome, and headache.

### Safety

Adverse events were consistent with the well-established AE profile of iSRLs (Table 4). In the CH-ACM-01 trial, commonly reported AEs included gastrointestinal (e.g., nausea, diarrhea, dyspepsia, abdominal pain/distension, flatulence, vomiting), neurological (e.g., headache, dizziness), and musculoskeletal events (e.g., arthralgia, back pain) [18]. In the CHIASMA OPTIMAL trial, treatment-emergent AEs with ≥ 5% incidence that were more common in the OOC group than in the placebo group were diarrhea, nausea, abdominal discomfort, vomiting, dyspepsia, blood glucose increase, sinusitis, osteoarthritis, cholelithiasis, urinary tract infection, large intestine polyp, and pain.

**Table 4:** Overview of AEs in CH-ACM-01 and CHIASMA OPTIMAL trials

	CH-ACM-01 (N = 155)	CHIASMA OPTIMAL (N = 56)	
		OOC (n = 28)	Placebo (n = 28)
TEAEs, n	1096	172	219
Patients with any TEAE, n (%)	134 (86.5)	28 (100.0)	27 (96.4)
Treatment-related TEAEs, n	267	40	41
Patients with any treatment-related TEAE, n (%)	90 (58.1)	18 (64.3)	15 (53.6)
SAEs, n	29	3	1
Patients with any SAE, n (%)	17 (11.0)	2 (7.1)	1 (3.6)
Treatment-related SAEs, n	3	0	0
Patients with a treatment-related SAE, n (%)	2 (1.3)	0 (0.0)	0 (0.0)
Mild TEAEs, n	691	118	150
Patients with maximum severity mild TEAEs, n (%)	31 (20.0)	11 (39.3)	8 (28.6)
Moderate TEAEs, n	316	46	56
Patients with maximum severity moderate TEAEs, n (%)	61 (39.4)	14 (50.0)	12 (42.9)
Severe TEAEs, n	84	8	13
Patients with maximum severity severe TEAEs, n (%)	42 (27.1)	3 (10.7)	7 (25.0)
TEAEs leading to study drug discontinuation, n	41	5	1
Patients with any TEAE leading to study drug discontinuation, n (%)	21 (13.5)	2 (7.1)	1 (3.6)
Treatment-related TEAEs leading to study drug discontinuation, n	34	5	0
Patients with a treatment-related TEAE leading to study drug discontinuation, n (%)	17 (11.0)	2 (7.1)	0 (0.0)
TEAEs leading to death, n	9	0	0
Patients with any TEAE leading to death, n (%)	2 (1.3)	0 (0.0)	0 (0.0)

*AE* adverse event, *SAE* serious adverse event, *TEAE* treatment-emergent adverse event

In both trials, the majority of events occurred within the first 3 months of treatment, with AEs decreasing over time. There were no dose-related AEs observed in the CH-ACM-01 trial. This assessment was not possible in CHIASMA OPTIMAL because of the small number of patients.

## Discussion

The results of the CH-ACM-01 and CHIASMA OPTIMAL trials demonstrated consistent results in biochemical response, durability of response, and preference to continue treatment with OOC, with safety profiles that are similar to iSRLs [18, 19]. The effect of OOC was notably preserved in both open-label (CH-ACM-01) and double-blind, placebo-controlled (CHIASMA OPTIMAL) trial designs. Both phase 3 trials have yielded unique information related to OOC as a treatment for acromegaly and provide information useful for clinicians treating acromegaly. The CH-ACM-01 trial enrolled a larger cohort of 155 patients, but CHIASMA OPTIMAL’s inclusion of a placebo control provides valuable insight into events that may be disease rather than treatment related.

These complementary trials enrolled similar acromegaly populations despite differences in inclusion criteria and the known variability of IGF-I levels [21, 25, 26]. Mean IGF-I levels at the start of the core treatment phase suggest that the enrolled populations are comparable between the 2 trials—for example, the cohort is representative of acromegaly patients who were previously biochemically responding to iSRLs. Periodic fluctuations above the ULN are common on SRLs, and the small difference in inclusion criteria did not appear to alter the phenotype of the patient population across the trials. Screening failures were slightly higher in CHIASMA OPTIMAL (53%) compared to CH-ACM-01 (34%), likely due to the more conservative entry criteria of IGF-I ≤ 1.0 × ULN. The focus of the current report is comparison of active OOC treatment; thus, data from the placebo arm of CHIASMA OPTIMAL [19] have been only briefly touched upon.

Determination of the most effective dose of OOC as it relates to the prior iSRL dose was also assessed in these trials. Despite the higher rate of patients receiving mid/high iSRL doses in CHIASMA OPTIMAL and variations in dose escalation, maintenance of response was similar in the two trials. Post hoc analyses based on prior SRL dose were performed solely on patients completing the CH-ACM-01 trial as responders (n = 82). Of OOC responders previously on a high dose of iSRLs, 45% finished the core trial on the lowest tested dose of OOC (40 mg), and 69% on low-mid doses of OOC (40–60 mg). In the CHIASMA OPTIMAL trial, it is difficult to extrapolate dose correlations in the same manner due to the limited sample size. Further complicating analysis, the dose titration in CHIASMA OPTIMAL trial was driven by predefined criteria in the protocol for trial withdrawal requiring participants to have been on the maximum dose, 80 mg, for 2 consecutive visits with an IGF-I ≥ 1.3 × ULN. In this manner, the dose escalation observed in CH-ACM-01 is more representative of standard clinical practice. Wear-off timing of previous long-acting iSRLs may influence dosing of OOC, as the placebo arm of CHIASMA OPTIMAL demonstrated a median of 16 weeks of continued control after stopping SRL injections [19], suggesting that a carryover effect of long-acting iSRLs may potentially delay need for OOC dose adjustments. However, neither of these trials could provide a clear indication of OOC dose based on prior iSRL dose.

Several currently approved iSRL therapies for acromegaly report outcomes using selective analysis in completers or less rigorous methods for handling missing data [27]. The 2 trials presented here assessed OOC efficacy using rigorous and conservative approaches to account for missing data, including LOCF (CH-ACM-01) and WOCF (CHIASMA OPTIMAL) as primary endpoints. Nevertheless, both trials showed consistent and effective maintenance of biochemical response in patients with acromegaly. For CH-ACM-01, an additional post hoc TWA, which may provide a more accurate and clinically meaningful assessment, showed an even greater response as compared with end-of-treatment analysis in the fixed-dose population at the end of the extension phase [14]. Additionally, 80% of the patients entering the fixed-dose phase in CH-ACM-01 maintained or improved acromegaly symptoms from baseline to the end of treatment. This was observed despite the majority of patients demonstrating biochemical response at baseline from prior iSRLs. The reduction in symptoms at the end of the trial was possibly due to more frequent capsule dosing that could result in more consistent circulating octreotide levels, helping to reduce breakthrough symptoms that are seen with the longer dosing intervals used with iSRLs.

In both trials, the majority of patients who were fully or partially responding at baseline maintained response at the end of the trial. Importantly, a portion of patients in CH-ACM-01 who were not biochemically controlled at baseline demonstrated biochemical response by the end of the trial; the single patient in CHIASMA OPTIMAL who had an IGF-I > 1.0 × ULN at baseline, improved by the end of the trial. These data indicate that treatment with OOC can maintain biochemical control of acromegaly and may additionally help achieve control in patients who have elevated IGF-I levels while receiving iSRLs. Patients should be closely monitored to ensure long-term biochemical control, as results from these trials highlight the variability of IGF-I levels in patients with acromegaly. However, for optimal dosing and maintenance of response, it is important that patients take OOC with water ≥ 1 h before a meal or ≥ 2 h after a meal. Importantly, if biochemical control was lost while a participant was receiving OOC, reversion to their previous iSRL dose in CHIASMA OPTIMAL resulted in regain of control within 4 weeks, or 1 injection cycle [19]. Among the patients who completed the dose adjustment period of the 2 trials and were stabilized on a fixed dose of OOC, most achieved a durable response and entered the voluntary extension phase. Notably, patient-reported symptoms should be considered in dose titration decisions, as symptoms reported by health care providers and symptoms reported by patients are often not congruent, particularly in regards to frequency, severity, and pattern of symptoms [28].

OOC were found to be safe and well tolerated in both trials. The most common TEAEs were gastrointestinal, neurological, and musculoskeletal AEs, and were consistent with the known safety profile of iSRLs. Furthermore, no dose-related AEs were observed. In the CHIASMA OPTIMAL trial, approximately double the number of AESIs were observed in the placebo group, including higher rates of arthralgia, hyperhidrosis, fatigue, and peripheral swelling, mirroring the loss of biochemical control observed in these patients after withdrawal of active treatment.

Despite disease control achieved with current iSRL options, there remains an unmet need for patients with acromegaly receiving regular injections due to treatment inconvenience, injection-related pain, and complications [5, 9, 13, 28]. Many patients with acromegaly desire an alternative to injections, in the form of an oral treatment, to alleviate some of these burdens [13]. The oral octreotide formulation precludes all possibility of injection site reactions that are commonly encountered with existing iSRLs. OOC represents a safe and effective treatment option for many patients with acromegaly who require medical therapy, allowing patients who are currently responding to iSRL therapy to be safely transitioned to oral therapy if they desire, with the potential to decrease the burden and costs associated with injections.

## Data Availability

The datasets generated during and/or analyzed during the current trial are available from the corresponding author on reasonable request.
