# Correction to: Oral octreotide capsules for the treatment of acromegaly: comparison of 2 phase 3 trial results

**DOI:** 10.1007/s11102-021-01177-w

**Published:** 2021-08-04

**Authors:** Artak Labadzhyan, L B Nachtigall, M Fleseriu, M B Gordon, M Molitch, L Kennedy, S L Samson, Y Greenman, N Biermasz, M Bolanowski, A Haviv, W Ludlam, G Patou, C J Strasburger

**Affiliations:** 1 Cedars-Sinai Medical Center, Los Angeles, CA 90048 USA; 2 MGH Neuroendocrine and Pituitary Center, Chestnut Hill, MA USA; 3 Pituitary Center, Oregon Health & Sciences University, Portland, OR USA; 4 Allegheny Neuroendocrinology Center, Allegheny General Hospital, Pittsburgh, PA USA; 5 Northwestern University, Chicago, IL USA; 6 Cleveland Clinic Foundation, Cleveland, OH USA; 7 Mayo Clinic, Jacksonville, FL USA; 8 Sourasky Medical Center and Tel Aviv University, Tel Aviv, Israel; 9 Leiden University Medical Center, Leiden, The Netherlands; 10 Wroclaw Medical University, Wroclaw, Poland; 11 Chiasma, Inc., Needham, MA USA; 12 Charite Universitatsmedizin, Berlin, Germany

## Correction to: Pituitary 10.1007/s11102-021-01163-2

The article ‘Oral octreotide capsules for the treatment of acromegaly: comparison of 2 phase 3 trial results’, written by Artak Labadzhyan, L B Nachtigall, M Fleseriu, M B Gordon, M Molitch, L Kennedy, S L Samson, Y Greenman, N Biermasz, M Bolanowski, A Haviv, W Ludlam, G Patou and C J Strasburger was originally published electronically on the publisher’s internet portal on 25 June 2021 without open access. With the author(s)’ decision to opt for Open Choice the copyright of the article changed on 15 July 2021 to © The Author(s) 2021 and the article is forthwith distributed under a Creative Commons Attribution 4.0 International License, which permits use, sharing, adaptation, distribution and reproduction in any medium or format, as long as you give appropriate credit to the original author(s) and the source, provide a link to the Creative Commons licence, and indicate if changes were made. The images or other third party material in this article are included in the article’s Creative Commons licence, unless indicated otherwise in a credit line to the material. If material is not included in the article’s Creative Commons licence and your intended use is not permitted by statutory regulation or exceeds the permitted use, you will need to obtain permission directly from the copyright holder. To view a copy of this licence, visit http://creativecommons.org/licenses/by/4.0.

The original article has been corrected.

